# Engaging stakeholders to develop a suicide prevention learning module for Louisiana firearm training courses

**DOI:** 10.1186/s40621-023-00413-0

**Published:** 2023-01-11

**Authors:** Claire Houtsma, Jeffrey Powers, Amanda M. Raines, Matthew Bailey, Catherine Barber, Gala True

**Affiliations:** 1 Southeast Louisiana Veterans Health Care System, 2400 Canal Street, New Orleans, LA 70119 USA; 2 South Central Mental Illness Research, Education and Clinical Center, New Orleans, LA USA; 3 Louisiana State University Health Sciences Center, New Orleans, LA USA; 4 Louisiana State University, Baton Rouge, LA USA; 5 Harvard T. H. Chan School of Public Health, Boston, MA USA

**Keywords:** Lethal means safety, Firearm owners, Firearm instructors

## Abstract

**Background:**

Firearm suicide is a significant public health problem in the United States of America among the general and veteran populations. Broad-based preventive strategies, including lethal means safety, have been emphasized as a key approach to suicide prevention. Prior research has identified ways to improve the reach and uptake of lethal means safety messages. However, few resources have been created with these lessons in mind.

**Methods:**

Louisiana firearm owners and instructors were recruited through a larger project, Veteran-Informed Safety Intervention and Outreach Network, as well as a publicly available database of firearm instructors to participate in focus groups to provide feedback on an existing suicide prevention learning module (developed in Utah) for use by firearm instructors. Their feedback was used to adapt the module, which included a brief video and PowerPoint presentation. Firearm owners and instructors were then invited back for another round of focus groups to provide feedback on this adapted learning module. Team-based rapid qualitative analysis was conducted to identify themes across transcripts from these four focus groups.

**Results:**

Firearm owners and instructors agreed on several key themes, including the importance of messenger relatability and aligning the lethal means safety message with firearm owner values. Feedback suggested these themes were adequately addressed in the adapted learning module and contributed to overall module acceptability. The final theme, present across the original and adapted learning modules (i.e., Utah and Louisiana), was openness to further information and training on firearm suicide prevention.

**Conclusion:**

Consistent with a public health approach to suicide prevention, the current study used stakeholder engagement to develop a suicide prevention learning module perceived as representative, accurate, and acceptable to Louisiana firearm owners and instructors. These findings can be used to inform firearm suicide prevention efforts in other states.

**Supplementary Information:**

The online version contains supplementary material available at 10.1186/s40621-023-00413-0.

## Background

Suicide is a pervasive public health concern in the United States of America (US) within both the general and veteran populations. As the 12th leading cause of death in the US (American Foundation for Suicide Prevention 2022), suicide accounts for over 45,000 deaths annually (Centers for Disease Control and Prevention, National Center for Injury Prevention and Control 2005). Although in previous decades veterans had a lower suicide rate than their demographic peers, more recently their suicide rate has significantly exceeded that of their demographic peers (Bullman and Schneiderman 2021). Additionally, firearms are the leading suicide method for both civilians and veterans, accounting for 52.8% of general population and 69.2% of veteran suicides (Centers for Disease Control and Prevention, National Center for Injury Prevention and Control 2005; Department of Veterans Affairs 2021). Notably, firearms have a case fatality rate of nearly 90% (Conner et al. 2019); and most who die by firearm suicide do so on their first attempt (Anestis 2016; Berrigan et al. 2022), suggesting a need for research and enhanced communication surrounding *preventive* strategies to reduce risk of firearm suicide.

Given that prediction of imminent risk for suicidal behavior has not significantly improved in the last few decades (Franklin et al. 2017), broad-based public health initiatives designed to reach and impact larger groups have been widely promoted (Anestis et al. 2017; National Action Alliance for Suicide Prevention, Lethal Means Stakeholder Group 2020; Adams and Dahlen 2021). One such approach is “lethal means safety,” which involves temporarily removing or limiting access to specific methods of suicide, particularly during times of crisis (Florentine and Crane 2010; Barber and Miller 2014; Bryan et al. 2011). Population-based studies have demonstrated the utility of limiting access to firearms before suicidal crises (e.g., storing away from home, or locked at home in such a way that an at-risk person has no access) (Sarchiapone et al. 2011; Reisch et al. 2013; Shelef et al. 2016). Indeed, lethal means safety and reducing access to firearms has been embraced in national suicide prevention strategies for the general (Office of the Surgeon General 2021) and veteran populations (United States Government 2021). Given that firearm ownership has been consistently linked to an increased risk of suicide death (Anestis and Houtsma 2018; Miller et al. 2016; Anglemyer et al. 2014), and that suicide is the leading type of firearm death, providing lethal means safety training in settings where firearm owners expect to hear safety messages about firearm use—such as introductory firearm classes—may be a promising place to begin.

Prior research has demonstrated the importance of stakeholder perspectives in effectively framing lethal means safety content to increase the reach and acceptability of such messages. Pallin and colleagues (Pallin et al. 2019) identified key themes that are important to framing lethal means safety messaging among firearm owners, as well as specific content that is preferred. For example, stakeholders indicated that it is important to acknowledge the strong safety culture within the firearm-owning community, emphasize firearm owners’ agency and responsibility in preventing suicide, and use credible messengers to communicate the information. Furthermore, research supports the involvement of stakeholders in community-based firearm suicide prevention efforts to amplify lethal means safety messaging (Henn et al. 2019).

Consistent with this emerging literature, Barber and colleagues (Barber et al. 2019) partnered with the Utah Firearm Safety Workgroup to add information on firearm suicide prevention and lethal means safety to the curriculum of the basic firearm safety course required of those seeking a Utah Concealed Firearm Permit. They adapted and shortened a generic Means Matter PowerPoint presentation for firearm instructors, piloted it with a small sample of firearm instructors, and then sent it out more broadly via survey for comment from close to 1,000 firearm instructors across the nation.^1^ Survey results indicated that two-thirds of instructors were interested in teaching this material (Barber et al. 2019) (see Table 1 for more information regarding this learning module). After finalizing the PowerPoint presentation, members of the Utah Firearm Safety Workgroup created a video to demonstrate how the information could be presented without slides (Schaeffer-Duffy 2018). In 2019, instructors in Utah were notified that the suicide prevention module was a required component of the curriculum.

This pioneering work had several limitations. Feedback on the module was not sought from the actual target of the material: students in firearm classes. Further, the video was largely intended as a tool for instructors, rather than an independent method for communicating module material. Given that the Utah video was created for a different purpose and used suicide statistics in Utah, it has limited relevance to firearm owners and instructors in other states. Therefore, the purpose of this study was to adapt and expand Utah’s learning module, in collaboration with local civilian and veteran firearm owners and instructors, to develop a culturally competent suicide prevention learning module that could be used in Louisiana firearm safety and concealed carry courses. Additionally, the current study aimed to assess the initial feasibility and acceptability of this learning module. It was expected that the adapted and expanded module would be considered feasible and acceptable to firearm owners and instructors.

## Methods

### Participants

Participants were firearm owners (*n* = 10) and firearm instructors (*n* = 8) recruited from a larger project, titled Veteran-Informed Safety Intervention & Outreach Network (VISION). VISION is part of a federally funded quality improvement project by the Department of Veterans Affairs (VA) aimed at reducing and preventing veteran suicide by firearm. VISION works with an active coalition of firearms professionals and instructors, law enforcement officers, veterans, community leaders, and health professionals to identify and/or develop strategies to prevent veteran suicide. Participants across focus groups ranged in age between 29 and 60 (*M* = 41.75, SD = 10.34) and were predominantly male (61.1%) and White (55.5%), with 38.9% identifying as Black/African American and 5.6% with missing data for race. In terms of ethnicity, 5.5% identified as Hispanic and 11.1% had missing data. The majority of participants identified as veterans (55.6%), with 40% having served in the Army, 20% in the Navy, 20% in the Air Force, 10% in the Marine Corps, and 10% in the Army National Guard. The number of years participants reported having owned a firearm ranged from 3 to 36 (*M* = 22.73, SD = 9.62), and 50.0% reported having a concealed carry permit.

### Procedure

#### Recruitment

The larger project under which the current project fell, VISION, was deemed a quality improvement project by the Southeast Louisiana Veterans Health Care System Institutional Review Board. To recruit firearm owner and instructor focus group participants, the authors first contacted known VISION coalition members. Of the 17 individuals contacted, seven firearm owners and two instructors agreed to participate. In addition, the authors consulted a publicly available list of concealed handgun permit instructors from the Louisiana State Police website (Louisiana State Police 2021) to inquire by telephone and email about interest in participating. The authors began by reaching out to instructors in southeast Louisiana, choosing names at random from the New Orleans, Lake Charles, and Lafayette regions. Of the six instructors contacted, five agreed to participate. Recruitment efforts ceased when both focus groups reached six to eight members. Those who agreed to participate were provided with a link to a virtual focus group meeting; separate groups were held with firearm instructors and firearm owners.

#### Focus groups

Four virtual focus groups were held over five months between May and October 2021. In advance of the focus groups, participants were informed that the group discussion would be recorded for evaluation purposes. Verbal consent was obtained from all participants prior to beginning focus groups. The first two focus group sessions were conducted in May 2021, one with five firearm owners and the other with four firearm instructors. These audio-recorded sessions lasted approximately 90 min. Following brief introductions, participants were shown the suicide prevention learning module developed by the Utah group (Barber et al. 2019). Specifically, participants were shown the brief video, followed by focus group questions and discussion, and then the PowerPoint presentation, also followed by focus group questions and discussion. The questions were designed to elicit feedback on likes and dislikes about the module, changes that would make it more relatable and Louisiana-specific, and elements that most effectively supplemented prior knowledge (see Additional file 1 for the focus group guides).

Using a rapid analysis approach described below, the authors incorporated input from the focus groups to develop an adapted video and updated PowerPoint presentation. Two veteran volunteers collaborated with the authors on the revised video script and served as the “presenters” in the finished video. Then, participants from the first focus groups were invited back (62.5% retention), and new participants were recruited. Focus group procedures were repeated for the second set of focus groups in October 2021, using the newly created module (i.e., adapted video and updated PowerPoint presentation) with one group of firearm owners (*n* = 8) and one group of firearm instructors (*n* = 6). Firearm owners and instructors who participated were compensated with a $75 gift card for each focus group they attended.

### Data analytic plan

Focus group discussions were transcribed verbatim and analyzed in two phases. In phase 1, two members of the research team coded the first two focus group transcripts, using a rapid qualitative analysis approach to identify elements of the Utah video and slides to retain, adapt, or revise (Lewinski et al. 2021). In phase 2, all four focus group transcripts were analyzed by three of the authors, using a team-based, iterative process as described in the Sort and Sift, Think and Shift approach (Maietta et al. 2021). This included a separate review of each transcript to develop an overview of participant views and opinions present within each group, and using PowerPoint to create an inventory of quotations for each focus group. Through team review and discussion, the quotations from all four focus groups were sorted into core categories to aid in the identification of themes relevant to the goal of providing acceptable and effective lethal means safety messaging and education in firearm courses.

## Results

During the first and second focus groups, firearm owners and instructors provided feedback on the original Utah learning module (Barber et al. 2019), and several themes emerged across groups. In response to this feedback, specific alterations were made in the adapted learning module. During the third and fourth focus groups, firearm owners and instructors provided feedback and reactions to the adapted module. These themes, alterations, and representative focus group quotes are described below and are summarized in Tables 1 and 2.

**Table 1:** Summary of adaptations to training materials

Component of LMS training	Original Materials	Maintained from Original	Revised from Original
Speaker characteristics and tone *Video*	Single speaker Older, White male Credible messenger (i.e., firearm owner and instructor) Traditional advocate (i.e., chair of Utah Sports Shooting Council—formal authority within firearm community) Instructional/lecture format Speaker is standing	Credible messengers (i.e., firearm owners)	Two speakers Diverse and inclusive in terms of gender and race/ethnicity Nontraditional advocate (i.e., veteran firearm owners—informal experts within firearm community) Conversational Speakers are sitting, moving, or relating to each other
Environment and visuals *Video* *PowerPoint*	Classroom setting only Firearms and storage cases displayed, some firearms with cable locks installed, in entirely static manner Two graphics that reinforce statistics, shown as cutaway from the speaker Two pictures Limited color	Firearms, cable locks, and storage cases are displayed Graphics that reinforce statistics are shown ––	Multiple, non-classroom settings, including shooting range Dynamic demonstration of recommended actions (i.e., disassembling firearms, installation of cable locks, locking firearms in storage case separate from ammunition, handing over firearms to someone else to hold) Graphics displayed concurrent with speaker, additional visual display of information including “warning signs” and “risk factors” Inclusion of images in different settings (i.e., individuals at home, families) to illustrate concepts 23 pictures Five different colors across slides
Content *Video and PowerPoint*	General information about firearm deaths including homicide and suicide State-specific statistics Lock, Limit, Remove (i.e., strategies to increase time and distance) Encourage treatment seeking Evidence/rationale for time and distance Suicidal crises are often brief 90% who survive suicide attempt do not go on to kill themselves Description of warning signs Analogy to holding keys to prevent drunk driving Emphasis on avoiding government mandates, firearm owners working together	General information about firearm deaths including homicide and suicide State-specific statistics Lock, Limit, Remove Encourage treatment seeking Evidence/rationale for time and distance Description of warning signs Analogy to holding keys to prevent drunk driving Emphasis on avoiding government mandates, firearm owners working together	Included specific statistics about veterans Included statistics around suicide methods Included national and state suicide prevention resources for veterans and civilians (e.g., locations of free cable locks) Personal experiences with suicide were added * Video*: both speakers shared personal encounters with suicide and/or experiences with friends and family in need of help * PowerPoint*: Incorporated a real story about a suicidal crisis and temporary means restriction
Length *Video PowerPoint*	5 min, 25 s 14 slides	––	6 min, 6 s 23 slides

**Table 2:** Representative quotes by themes

Messenger needs to be relatable	*Original Materials* “It kind of felt like you were being lectured to—almost like fussed at.” “I think we should also have videos with women teaching so that there is not this stereotype or understanding that it is all men that are gun owners.” “You might need three different people…a Cajun guy for South Louisiana, somebody with a New Orleans accent for Southeast, and then a redneck for North Louisiana.” “bringing in other people like not only having him alone” “without sounding expert-ish and just kind of meeting people where they are? They tend to be more relatable…I can be an expert, but I also have to kind of know my audience.” “Somebody who’s very passionate…about the subject or have had close relations to an event like this would really bring in I think that charisma.”
	*Adapted Materials* “I liked how the group was not just Jim and Roy in the corner talking but was both men and women. That was well done because I think veterans are a diverse group of people.” “They felt like they were talking more to you and not at you.” “I felt like a connection with them…these are real people. This is real life…I actually feel what they are saying.” “It started off robotic, but that’s because they are real people…Even though that’s a critique, it’s important to leave that in there because you can tell they aren’t actors.” “They certainly sounded more local.” “He had the semicolon tattooed on his inner arm. That signifies he has survived an attempt, and it was good that they cut into that to where you could see it.” “That is a relatable story. I can assure you it has happened tons and tons of times in reality. I would not change it a bit.”
Message needs to align with firearm owner values	*Original Materials* “We had a family that needed to remove their firearms…we stored it for them until they felt like he was better…that was just because they felt like we were trustworthy. We were honest. We weren’t going to spread their information. We were there to help.” “two things that stood out on my end was the responsibility being on the family and the friends, and the community.” “everybody has a…clear cut line and personal sense of freedom. And the guy kind of wrapped it up at the end saying… ‘We can do this together and without government mandates’.” “makes you as a gun owner who’s sitting through a class like that feel somewhat responsible, meaning you may want to be conscious and aware of what, you know, people who are around you are going through…it’s not just about self-defense or protection when it comes to firearms.”
	*Adapted Materials* “I’m kind of deep in the gun nerd stuff. So, I kind of brush elbows with people that are not going to listen to anything you say the minute they feel like one, you don’t know anything about firearms or two, that it’s pushing you toward the all-feared gun control point. I think, one, showing these people shooting and using real hardware actually a nice point that might grab some of those people and say, okay, maybe this is not made by people that have no idea what they’re talking about. Two, there was a line… ‘we can do this without government mandates’. I think for a lot of people in the gun community…that’s a big thing.” “…that little bit of message there at the end about doing it ourselves rather than being government mandated, I think that is also an important message…This is one fight we can all get behind. We can all agree on. We can all work together to help support.”
Desire for more	*Original Materials* “maybe add the different…security methods.” “I think with the mental health it has to be broken up a little bit, or scenarios have to be given…there also needs to be examples of a crisis.” “breaking it off into different sections…a section for dealing with mental health…a section for dealing with communication…how to safely take something from someone, or to safely call.” “say I have identified that my neighbor possibly may be suicidal. How do I approach them? How do I start the conversation about holding their guns? How, you know, do I not trigger them into doing something even worse?…How do I start up this conversation and lead it in a good direction, that way I’m more helpful than I am harmful? “…you could break it down a little bit further with adults and teenagers and children…”
	*Adapted Materials* “If there was a way like just clear contact information…if you really need to get out of the situation right now, call this number. Go to this place…try to make that information readily available for somebody who at a time of crisis might just have the one second of seeing it.” “I think that there should be a list of sayings of like how one can ask a friend to or a loved one to hold their firearms. Or how, if you know someone is at risk, how you can ask them without being judgmental…examples of things that you could say without disclosing too much personal information or without becoming judgmental.” “Toward the end of the video I think there should be kind of a little guide on how to interact…having a little slide where the location where someone could store—let us say if the person stays in the same household. Where is an alternative source where they could store it?”
Acceptability	*Original Materials* “I would probably use a more engaging video. And then, send home this PowerPoint more in a pamphlet version” “I wouldn’t be opposed to adding anything that would kind of join in the culture today…” “I think you guys can touch more people…by bringing this information to outreach program, and community safety watch meetings…not just bringing this to gun owners themselves, touching everybody that deals with suicide might actually be a lot more beneficial.” “I’m not sure that the firearm instructor level is the most effective arm to get this information out because I think especially in Louisiana…folks go to those because they have to…the entire time they just can’t wait to leave.” “PowerPoint seems to be the easiest to control and translate” “I think a little bit of both helps [PowerPoint and video]”
	*Adapted Materials* “I think this was an impressive video. You hit all of the points that the first group meeting discussed about what would improve upon the video that we saw produced in Utah.” “I want to say bravo. You guys did a great job. That is literally night and day from the last video.” “the statistics were great.” “I think it’s important that you guys put real people in it…I think relating it to a person like that transition from informational to real people I think is a very good, good methodology.” “It looked well produced. As far as the message is concerned, it was not much about beating around the bush. It gave good solid information…these are the statistics of what happens. These are some fool-proof methods to help prevent it from happening.” “I personally do not have a problem with it…I think it will connect with the audience in a way that makes it believable and that is receptive. It was not too long. It was not too short.” “It made it where my students, if I were to show it to them, they would feel like it is an easy thing to step into and be able to accomplish.” “I would say just the video…the minute the PowerPoint slips on for me, we’re in death by PowerPoint mode. I start fading fast.” “a video would be a nice break and they might actually…pay more attention, honestly.” “I would use both. I would use some of the slides. I definitely would play the video during breaks. I can already see how that would work out really well.” “The video is a really easy medium to get the information from the slides across in a way that we do not have to sit there and click across multiple slides.”

### The messenger needs to be relatable

Firearm owners and instructors reported that the messenger in the video who was presenting the Utah learning module was too directive and lacked emotionality. There was also a call for diversity, representation of the local culture, and multiple messengers. One firearm instructor disagreed, indicating there may not be a significant benefit in making the video specific to Louisiana, and believing that experts in firearm training would be the most preferred messengers. However, most instructors echoed the sentiments of the firearm owners, indicating a preference for multiple speakers and a messenger who could balance expert knowledge with emotion and relatability. In response, we incorporated several changes in the adapted learning module. First, we recruited two local firearm-owning veterans from diverse gender, racial, ethnic, and military service backgrounds to serve as the messengers in our video. These veterans were a Latino man who served in the Marine Corps and an African American woman who served in the Army. Preproduction interviews were conducted with these individuals to understand their motivations for participating, learn about personal experiences relevant to firearm suicide prevention, and identify suicide prevention messages that were particularly important to them. These features were then incorporated into the video script to enhance the emotional salience and personal relevance of the messages. In addition, we added a personal story based on true events to the module PowerPoint slide deck to highlight the emotional salience and personal relevance of firearm suicide prevention in this alternative presentation format (see Table 1 for additional changes made to the learning module). Firearm owners and instructors responded very well to these changes in the adapted learning module. Both groups felt the messengers were relatable and representative of Louisiana firearm owners. Focus group participants who viewed the adapted module reported that the emotional salience and personal relevance of the message were clear. The main suggestion for further improvement was to make the personalized story within the PowerPoint into a video: “It’s a heartfelt story that I don’t know if it translates well to slides.” Unfortunately, this modification was not feasible given limited resources.

### The message needs to align with firearm owner values

Focus group participants agreed on the importance of highlighting firearm owner responsibility in firearm suicide prevention: specifically, that firearm owners have a responsibility to their friends, families, and fellow firearm owners to be aware of the risk of firearm suicide and offer assistance to prevent this outcome. There was also broad agreement with the notion that this can be accomplished “without government mandates.” These were two messages included in the Utah learning module that were maintained in the adapted materials, given their broad acceptability across focus groups. One instructor felt the language surrounding the message of “responsibility” should be altered slightly to preserve firearm owner autonomy:

“We have a responsibility to provide an option for our friends and family…If that friend or that other fellow gun owner goes ‘hey man, no. I do not want to do this. No, you cannot take my firearm.’ There needs to be a pretty clear-cut line there that says that is your decision to make.”

A firearm owner also raised concerns regarding the relevance of firearm retailers as a resource for veterans, as follows:

“There seems to be a misconception of…veterans are more buddy-buddy with the gun stores than they actually are…you’re going to have better luck focusing on talk to your battle buddies, the people that you served with, your veterans in your community…”

These points were addressed by adjusting language on modifiable content (i.e., PowerPoint slides) and demonstrating the option of asking a friend to hold on to a firearm temporarily (i.e., one veteran hands off a firearm to another in the video). Focus group participants who viewed the adapted module echoed approval of the aforementioned messages and further highlighted that accompanying visuals (e.g., veterans shooting firearms at the range) served to bolster the legitimacy of these messages. No further edits were recommended relevant to this theme.

### Openness to further information and training

Firearm owners and instructors indicated a desire for more information, resources, and training in firearm suicide prevention. Namely, participants who viewed the original module reported interest in learning how to communicate directly with someone who may be at risk of firearm suicide and requested more concrete resources for individuals who may be looking for places to store their firearms in a crisis situation. Finally, there was a request for more detailed information in several content areas (e.g., examples of storage methods, mental health crises, and suicide statistics for teens/children). Some of the requests for detailed information could be incorporated (e.g., examples of storage methods and resources). However, others would have considerably lengthened the learning module, such as the request for examples of ways to engage in a conversation about suicide risk and out-of-home storage, and were therefore not incorporated in the adapted version. Focus group participants who viewed the adapted module reiterated two areas of interest for continued training: how to engage those at risk for suicide in a conversation about firearm storage and information about out-of-home storage options. Given time and resource constraints, we were unable to incorporate examples of conversations about firearm storage. However, we did choose to incorporate an additional slide (in the video and PowerPoint presentation) with a website link and map of local out-of-home storage partners in Louisiana.

### Acceptability

Participants who viewed the original learning module reported lukewarm perceptions of acceptability. Most found it to be acceptable; however, several expressed views that firearm instructors may not be the appropriate channel through which to communicate such information. There were also conflicting views about which medium was most effective (i.e., video versus PowerPoint). Unanimously, firearm owners and instructors who viewed the adapted module believed it to be acceptable. Those who viewed both the original and adapted modules highlighted many of the aforementioned changes to the original module as positive improvements that increased acceptability. Firearm instructors indicated they would likely use a combination of both the video and the PowerPoint presentation in their courses, whereas firearm owners expressed the most interest in receiving the video if they were taking a firearm safety or concealed carry course. There were some additional critiques of the adapted learning module, “With it being a suicide-centered video, I think it could probably use less gun handling in it. Suicide by gun is not just a gun person thing,” and “Make sure the shooting side is competent [in the video].” To address these remaining concerns, the authors cut some video footage that displayed incorrect shooting stance or grip. The majority of the focus group participants who viewed the adapted module felt the visuals, speakers, and content were compelling and acceptable.

## Discussion

The purpose of the current study was to collaborate with local firearm owners and instructors to adapt and expand Utah’s learning module (Barber et al. 2019) to develop a culturally competent suicide prevention learning module that could be used in Louisiana firearm safety and concealed carry courses. A secondary purpose of this study was to assess the feasibility and acceptability of such a module among local firearm owners (the target audience in a firearm course) and instructors. Focus group feedback indicated a desire for relatable and representative messengers, and messages that align with firearm owner values (e.g., safety and responsibility). Following the development of the adapted module, focus group feedback suggested that participants found the module to be relatable, feasible, and acceptable.

Focus group feedback highlighted the value of using credible, relatable messengers. Focus group members viewed veterans as excellent messengers in the adapted learning module, indicating that they came across as knowledgeable, passionate, and genuine. This feedback aligns with previous research indicating that firearm owners and non-firearm owners view military veterans as highly credible messengers about firearm safety for suicide prevention (Anestis et al. 2021; Crifasi et al. 2018). Thus, the current study built upon the Utah learning module’s foundation by incorporating veteran perspectives in the adaptation and presentation of our learning module, which appears to have had a positive effect on module acceptability. Harnessing veteran perspectives and voices in this area may be particularly important, as veterans are more likely to own firearms (Lambert and Fowler 1997) and use less safe firearm storage practices (Anestis et al. 2020; Bryan et al. 2019; Simonetti et al. 2019) than individuals in the general population. The current study also highlighted the importance of other aspects of the messenger that augment credibility, including representation of multiple groups (e.g., women), incorporation of emotional closeness to the issue (e.g., suicide loss survivor, general passion for suicide prevention), and messenger tone (e.g., conversational vs lecturing). Furthermore, elements of the visual experience proved to be important to messenger credibility and overall acceptability, such as using correct firearm technique in video sequences involving shooting.

Importantly, prior research has highlighted the importance of incorporating certain themes into lethal means safety messaging, such as acknowledging the strong safety culture within the firearm-owning community and emphasizing firearm owners’ agency and responsibility in suicide prevention (Pallin et al. 2019). Findings from the current study support this perspective, as focus group members favored messaging that emphasized firearm owners’ responsibility to be knowledgeable about the risks of firearm suicide. Several motivations for such messaging were salient among firearm owner and instructor focus group members, including concern for the welfare of friends and family members, as well as the importance of addressing problems without government involvement. These messages were consistent with the Utah learning module and shed light on two potentially important values within firearm culture: personal accountability and autonomy. Accordingly, many firearm owners and instructors expressed a desire for more information, resources, and training in suicide prevention to support the preference to decrease firearm suicide through individual-level actions.

As with any study, several limitations should be noted. The suicide prevention module developed during the current study was tailored to an audience within Louisiana. Thus, the messages and associated impact of this module may not generalize to audiences in other states. Also of note, although firearm suicide is a substantial problem in Louisiana, firearm homicide accounts for a greater proportion of firearm-related deaths in this state (Centers for Disease Control and Prevention, National Center for Injury Prevention and Control 2005). As such, the module may be less impactful in Louisiana than in most other states where firearm suicide accounts for the majority of firearm-related deaths. Further, a limited number of participants provided feedback on the perceived acceptability of the module, so these views may not be representative of all Louisiana firearm owners and instructors. Relatedly, many participants in the current study were recruited from VISION, a project focused on Veteran firearm suicide prevention. As a result, there may have been a selection bias that influenced some participants’ views on the module, which may further limit the generalizability of our findings. Additionally, those who participated in the first round of focus groups (during which the Utah module was shown) were invited back for the second round of focus groups (during which the adapted module was shown). Although engaging participants in iterative focus groups is an acceptable qualitative research method (e.g., Bennett et al. 2020), it may have led to bias, given that these individuals had an additional point of reference when viewing the adapted module. Similarly, providing suggestions for improving the module during the first focus group may have led some participants to perceive personal investment in the resulting product, thereby biasing their feedback on the adapted module. Finally, the current study did not assess the efficacy of the adapted learning module on student outcomes. Thus, we cannot conclude whether the current module improves knowledge surrounding firearm suicide or impacts firearm storage-related behavior change.

Previous research has highlighted the importance of incorporating numerous stakeholder perspectives to craft culturally competent and credible firearm safety messages (Pallin et al. 2019; Barber et al. 2019). More recently, a literature review identified incorporating lethal means safety education into concealed carry curricula as a major opportunity for intervention to decrease veteran suicide (Consolino and Yarvis 2022). This study answers that call and builds on previous literature by engaging firearm owners and instructors, both veteran and non-veteran, in developing a suicide prevention learning module that was viewed as representative, accurate, and acceptable to Louisiana firearm owners and instructors. Given our limited ability to identify those at risk for suicide prior to an attempt (Franklin et al. 2017), it is critical to reach firearm owners with information about firearm suicide prevention on a large scale, regardless of acute suicide risk. Thus, this module is consistent with the broad public health approach to suicide prevention that is promoted by the US government (Office of the Surgeon General 2021; United States Government 2021). Although the module developed in this study was designed with Louisiana concealed carry classes in mind, there are other possible outlets for dissemination (e.g., law enforcement agencies, mental health outreach events, hunter safety courses, and firearm conferences), which could facilitate even greater reach. Indeed, the lethal means safety and suicide prevention strategies discussed in the module are applicable on a national level and could be used outside the context of Louisiana concealed carry courses.

Future studies should examine the impact of this learning module on relevant outcomes (e.g., firearm storage practices, knowledge about firearms and suicide) to determine its utility in suicide prevention efforts. Indeed, the authors are currently collaborating with Louisiana firearm instructors, collecting data with students in their concealed carry classes, and examining the effectiveness of this learning module on outcomes such as openness to changing firearm storage practices, knowledge about firearms and suicide, and actual behavior change following exposure to the learning module. If research supports the utility of this learning module, it may represent a critical resource for Louisiana firearm instructors interested in preventing firearm suicide. As previously noted, this module may be effective on a national level and outside the context of concealed carry courses. Therefore, another important consideration for future research is determining the various contexts in which this module is acceptable and effective. The learning module, including the brief video and downloadable PowerPoint slide deck, can be found on the VISION website (True 2022).

## Conclusions

In summary, the current study used stakeholder engagement to develop a suicide prevention learning module that was perceived as representative, accurate, and acceptable to Louisiana firearm owners and instructors. These findings support the public health approach to suicide prevention by introducing an acceptable form of training that can be disseminated widely to firearm owners, even in the absence of current suicide risk. Such interventions may prove vital to successful firearm suicide prevention efforts and should be further evaluated for effectiveness.

## Supplementary Information

**Additional file 1:** Focus Group Guides. Guides, including questions and prompts, used by the focus group facilitators. This document contains the guide used for each of the four separate focus groups.

## Data Availability

Data are available upon reasonable request from the first author (CH). Data include deidentified focus group transcripts.

## Abbreviations

US: United States of America
VA: Department of Veteran Affairs
VISION: Veteran-Informed Safety Intervention and Outreach Network

## Footnotes

1 The Utah concealed carry permit is valid in 37 states; as a result, two-thirds of instructors certified to teach the class that is required before obtaining a Utah Concealed Firearm Permit are in states other than Utah.
