# Supplementary material for: Engaging stakeholders to develop a suicide prevention learning module for Louisiana firearm training courses
Source: Inj Epidemiol. 2023 Jan 11;10:3. doi: 10.1186/s40621-023-00413-0 (PMC9832758; doi:10.1186/s40621-023-00413-0)
Supplement: Supplementary file 1 — Additional file 1: Focus Group Guides. Guides, including questions and prompts, used by the focus group facilitators. This document contains the guide used for each of the four separate focus groups. [file 40621_2023_413_MOESM1_ESM.docx]

**Focus Group Guide (Firearm Owners, Original Materials) – 90 minutes**

**I. Introduction (15 minutes)**

- Remind about recording
  - “as we discussed ahead of time, I am going to audio record our conversation so I can capture everyone’s words and be able to listen to and talk with everyone without having to take notes. The information that each of you share will be analyzed and will not be associated with any of you personally in any way. The recorder is turned on now, but you can ask me to stop or pause it at any time as your participation is completely voluntary.”
- Review Purpose of focus group
  - “we have asked each of you here today because you are experts on firearm ownership. We have some materials we hope can be helpful to include in firearm training; however, we need your honest opinions and feedback to make sure they are accurate and acceptable to Louisiana firearm owners”
- Allow group members to introduce themselves
  - “we’d like each of you to offer a brief introduction: name, occupation or important life role, and the primary reason why firearms are a part of your life (e.g., hunting, protection)”
- Framing for next 90 minutes
  - We are going to show a 5-minute video clip, followed by group discussion
  - Then, we will show PowerPoint slides followed by group discussion
  - We are looking for your honest feedback and have a few specific questions we’d like you to answer
  - We expect a range of opinions and anticipate we won’t all agree on everything – that is ok!
  - We ask for others to be respectful of others and to allow everyone the opportunity to share their thoughts
  - In the spirit of respecting one another, we also ask that opinions shared in this focus group stay in this focus group.
  - Questions?

**II. Video (30 minutes)**

- Show video
- Questions:
  - What did you like about the video? What did you dislike?
  - What changes would be important to make it Louisiana-specific? What other changes would you propose?
  - What was most compelling or best supplemented information you already knew?
  - Who should be presenting in the video?

**III. Slides (30 minutes)**

- Show PowerPoint slides
- Questions:
  - What was your impression of the slides?
  - What changes would be important to make it Louisiana-specific? What other changes would you propose?
  - What was most compelling or best supplemented information you already knew?

**IV. General Questions and Wrap Up (15 minutes)**

- How would you prefer to learn this information (i.e., video, slides, or both)? Which would you respond better to if you were in a training course?
- Thank you all for your time! We will take this information and use it to create materials that are a better fit for Louisiana firearm owners. We would be thrilled to have each of you back to provide feedback on the adapted materials. We will reach out to schedule this in a few months.

**Focus Group Guide (Firearm Instructors, Original Materials) – 90 minutes**

**I. Introduction (15 minutes)**

- Remind about recording
  - “as we discussed ahead of time, I am going to audio record our conversation so I can capture everyone’s words and be able to listen to and talk with everyone without having to take notes. The information that each of you share will be analyzed and will not be associated with any of you personally in any way. The recorder is turned on now, but you can ask me to stop or pause it at any time as your participation is completely voluntary.”
- Review purpose of focus group
  - “we have asked each of you here today because you are experts on firearm training. We have some materials we hope can be helpful to include in firearm training; however, we need your honest opinions and feedback to make sure they are accurate and acceptable to Louisiana firearm instructors”
- Allow group members to introduce themselves
  - “we’d like each of you to offer a brief introduction: name, occupation or important life role, and the primary reason why firearms are a part of your life (e.g., hunting, protection)”
- Framing for next 90 minutes
  - We are going to show a 5-minute video clip, followed by group discussion
  - Then, we will show PowerPoint slides followed by group discussion
  - We are looking for your honest feedback and have a few specific questions we’d like you to answer
  - We expect a range of opinions and anticipate we won’t all agree on everything – that is ok!
  - We ask for others to be respectful of others and to allow everyone the opportunity to share their thoughts
  - In the spirit of respecting one another, we also ask that opinions shared in this focus group stay in this focus group.
  - Questions?

**II. Video (30 minutes)**

- Show video
- Questions:
  - What did you like about the video? What did you dislike?
  - What changes would be important to make it Louisiana-specific? What other changes would you propose?
  - What was most compelling or best supplemented your existing training?
  - Who should be presenting in the video?

**III. Slides (30 minutes)**

- Show PowerPoint slides
- Questions:
  - What was your impression of the slides?
  - What changes would be important to make it Louisiana-specific? What other changes would you propose?
  - What was most compelling or best supplements information you already present during training?

**IV. General Questions and Wrap Up (15 minutes)**

- Would you be willing to include training on this topic in your courses? What barriers would you face? What would help you with implementing this?
- How would you prefer to present this information (i.e., video, slides, or both)? Why?
- Thank you all for your time! We will take this information and use it to create materials that are a better fit for Louisiana firearm owners and instructors. We would be thrilled to have each of you back to provide feedback on the adapted materials. We will reach out to schedule this in a few months.

**Focus Group Guide (Firearm Owners, Adapted Materials) – 90 minutes**

**I. Introduction (15 minutes)**

- Remind about recording
  - “as we discussed ahead of time, I am going to audio record our conversation so I can capture everyone’s words and be able to listen to and talk with everyone without having to take notes. The information that each of you share will be analyzed and will not be associated with any of you personally in any way. The recorder is turned on now, but you can ask me to stop or pause it at any time as your participation is completely voluntary.”
- Review Purpose of focus group
  - “we have asked each of you here today because you are experts on firearm ownership. Some of you saw the original materials during the first focus group. This included about 13 PowerPoint slides and a brief video about firearm suicide prevention. The feedback we received indicated that you all felt the statistics were powerful attention grabbers, there needed to be a more personal connection to suicide loss (e.g., a story), and that multiple, relatable and genuine speakers would improve the video. We took this feedback and incorporated it into adapted materials that we would like to show you today. We need your honest opinions and feedback to make sure the adapted slides and video are accurate and acceptable to Louisiana firearm owners”
- Allow group members to introduce themselves
  - “we’d like each of you to offer a brief introduction: name, occupation or important life role, and the primary reason why firearms are a part of your life (e.g., hunting, protection)”
- Framing for next 90 minutes
  - We are going to show the new 6-minute video clip, followed by group discussion
  - Then, we will show the new PowerPoint slides followed by group discussion
  - We are looking for your honest feedback and have a few specific questions we’d like you to answer
  - We expect a range of opinions and anticipate we won’t all agree on everything – that is ok!
  - We ask for others to be respectful of others and to allow everyone the opportunity to share their thoughts
  - In the spirit of respecting one another, we also ask that opinions shared in this focus group stay in this focus group.
  - Questions?

**II. Video (30 minutes)**

- Show video
- Questions:
  - What did you like about the video? What did you dislike?
  - What changes would you propose?
  - What was most compelling or best supplemented information you already knew?
  - How did you feel about the speakers?

**III. Slides (30 minutes)**

- Show PowerPoint slides
- Questions:
  - What was your impression of the slides?
  - What changes would you propose?
  - What was most compelling or best supplemented information you already knew?

**IV. General Questions and Wrap Up (15 minutes)**

- How would you prefer to learn this information (i.e., video, slides, or both)? Which would you respond better to if you were in a training course?
- Thank you all for your time! We will take this information and use it to create materials that are a better fit for Louisiana firearm owners.

**Focus Group Guide (Firearm Instructors, Adapted Materials) – 90 minutes**

**I. Introduction (15 minutes)**

- Remind about recording
  - “as we discussed ahead of time, I am going to audio record our conversation so I can capture everyone’s words and be able to listen to and talk with everyone without having to take notes. The information that each of you share will be analyzed and will not be associated with any of you personally in any way. The recorder is turned on now, but you can ask me to stop or pause it at any time as your participation is completely voluntary.”
- Review purpose of focus group
  - “we have asked each of you here today because you are experts on firearm training. Some of you saw the original materials during the first focus group. This included about 13 PowerPoint slides and a brief video about firearm suicide prevention. The feedback we received indicated that you all felt the statistics were powerful attention grabbers, there needed to be a more personal connection to suicide loss (e.g., a story), and that multiple, relatable and genuine speakers would improve the video. We took this feedback and incorporated it into adapted materials that we would like to show you today. We need your honest opinions and feedback to make sure the adapted slides and video are accurate and acceptable to Louisiana firearm instructors”
- Allow group members to introduce themselves
  - “we’d like each of you to offer a brief introduction: name, occupation or important life role, and the primary reason why firearms are a part of your life (e.g., hunting, protection)”
- Framing for next 90 minutes
  - We are going to show the new 6-minute video clip, followed by group discussion
  - Then, we will show the new PowerPoint slides followed by group discussion
  - We are looking for your honest feedback and have a few specific questions we’d like you to answer
  - We expect a range of opinions and anticipate we won’t all agree on everything – that is ok!
  - We ask for others to be respectful of others and to allow everyone the opportunity to share their thoughts
  - In the spirit of respecting one another, we also ask that opinions shared in this focus group stay in this focus group.
  - Questions?

**II. Video (30 minutes)**

- Show video
- Questions:
  - What did you like about the video? What did you dislike?
  - What changes would you propose?
  - What was most compelling or best supplemented your existing training?
  - How did you feel about the speakers?

**III. Slides (30 minutes)**

- Show PowerPoint slides
- Questions:
  - What was your impression of the slides?
  - What changes would you propose?
  - What was most compelling or best supplements information you already present during training?

**IV. General Questions and Wrap Up (15 minutes)**

- Would you be willing to include training on this topic in your courses? What barriers would you face? What would help you with implementing this?
- How would you prefer to present this information (i.e., video, slides, or both)? Why?
- **PILOT**: we would like to assess the reaction of students to this training material. Would you be open to a follow-up conversation about piloting these materials in your firearm training course?
- Thank you all for your time! We will take this information and use it to create materials that are a better fit for Louisiana firearm owners and instructors.
